# *F*-squares over cellular automata

**DOI:** 10.1371/journal.pone.0355562

**Published:** 2026-08-12

**Authors:** Joanne Hall, Kerri Morgan, Stella Stylianou, Vindya Warnakulasooriyage

**Affiliations:** School of Science, RMIT University, Melbourne, Victoria, Australia; University of Milano–Bicocca: Universita degli Studi di Milano-Bicocca, ITALY

## Abstract

*F*-squares are a class of arrays in which each element occurs the same number of times in every row and column; they play a significant role in experimental design. This paper examines the conditions under which types of *F*-squares can be generated by cellular automata (CAs). The approach is based on analysing the row and column sums of the arrays produced by CAs over ${𝔽}_{2}$, recognising that equal sums are a defining characteristic of *F*-squares. Particular attention is given to pairs of columns whose binary representations differ only in their final bit, and to pairs of rows whose binary representations differ only in their first bit, with the aim of identifying local rules that ensure equal column sums and equal row sums. Two families of local rules are identified that satisfy these conditions: bipermutive local rules and constant local rules. These results reveal that every *F*-square generated by a CA over ${𝔽}_{2}$ is either a Latin square or a trivial *F*-square.

## 1 Introduction

The concept of *F-squares* is a generalisation of Latin squares, first introduced by MacMahon [1] in 1898 under the name “quasi-Latin squares.” Subsequently, *F*-squares have been examined both directly and indirectly by Finney [2–4], Freeman [5], and Addelman [6] as a secondary outcome of their research interests in experimental designs. The first formal definition and use of the term “*F*-square” is due to Hedayat, who also established foundational results on their properties, motivated by practical and theoretical needs in the theory of designs [7,8].

Let $\lambda_{1},\lambda_{2},...,\lambda_{s}$ be positive integers and $n=\sum_{i=1}^{s}\lambda_{i}$. A frequency square or more concisely an *F*-square, of type $\left(n;\lambda_{1},\lambda_{2},...,\lambda_{s}\right)$ and order *n* is an n×n array over {1,2,...,s} such that the each symbol i∈{1,2,...,s} appears exactly $\lambda_{i}$ times in each row and in each column. In the case where $\lambda_{1}=\lambda_{2}=...=\lambda_{s}=\lambda$, the square is said to be type (n;λ) [9]. An *F*-square of type (*n*; 1) corresponds to a *Latin square* of order *n*. When the symbols {1,2,...,s} represent treatments, a square of type $\left(n;\lambda_{1},\lambda_{2},...,\lambda_{s}\right)$ serves as an experimental design with the properties: (a) treatment effects are orthogonal to row effects; (b) treatment effects are orthogonal to column effects; and, if $\lambda_{1}=\lambda_{2}=\cdots =\lambda_{s}=\lambda$, then (c) treatment arrangement is balanced within rows and columns; and (d) row effects are orthogonal to column effects [8]. There are two main reasons for considering *F*-square designs over Latin square designs in experimentation. First, when the difference between some treatments considered in previous experimentation as distinct is negligible and could be ignored. Second, when the number of treatments is smaller than the order of the square.

Recent research highlights a wide range of applications of *F*-squares in experimental design. Aggarwal et al. have explored mixture designs in orthogonal blocks using *F*-squares [10], nearly optimal orthogonally blocked designs for four mixture components based on *F*-squares [11], orthogonally blocked mixture component–amount designs via projections of *F*-squares [12], and optimal orthogonal block designs for four-mixture components in two blocks, based on *F*-squares for Becker’s models and K-models [13]. Husain et al. have further investigated optimal orthogonal designs in two blocks based on *F*-squares for the mixture inverse model with four components [14], *F*-square-based four-component *D*-, *A*-, and *E*-optimal orthogonal designs for an additive quadratic mixture model [15], uniform designs based on *F*-squares [16], and *F*-square-based optimal designs for reduced cubic canonical models with four components [17].

Research in the theory of *F*-squares primarily centres on constructing sets of mutually orthogonal *F*-squares (MOFS). Two *F*-squares, *F*_1_ and *F*_2_, of type $\left(n;\lambda_{1},\lambda_{2},...,\lambda_{s}\right)$ are said to be orthogonal if each ordered pair (*i*, *j*) occurs $\lambda_{i}\lambda_{j}$ times when the squares are superimposed. A MOFS is a set of *F*-squares in which each pair is orthogonal. A set of MOFS of cardinality *k*, each of type (n;λ), is denoted by *k*-MOFS(n;λ). Hedeyat et al. [18] showed that a set of type *k*-MOFS(n;λ) contains at most $k=(n-1{)}^{2}/(\frac{n}{\lambda }-1)$ squares. Such a set is called complete. The only complete set of MOFS known are of type (n;λ) with either: (i) *n* is a prime power; or (ii) if there exits a Hadamard matrix of order *n* for λ=2 [9]. Mavron [19] described complete sets of MOFS based on a technique for constructing affine 2-designs with parallel classes of lines. A set $\left\{F_{1},F_{2},...,F_{k}\right\}$ of *k*-MOFS(n;λ) is said to be maximal, denoted by *k*-maxMOFS(n;λ), if there does not exist a *F*-square $F_{j}$ of type (n;λ) that is orthogonal to every $F_{i}$ for all 1≤i≤k and i≠j. For even *n*, let μ(n) be the smallest *k* such that there exists a set of *k*-$\max\text{MOFS}(n;\frac{n}{2})$. It was shown in Britz et al. [20] that μ(n)=1 if $\frac{n}{2}$ is odd and μ(n)>1 if $\frac{n}{2}$ is even. This result was extended by Cavenagh et al. [21] who showed if $\frac{n}{2}$ is even, then μ(n)>2.

This paper investigates the conditions under which types of *F*-squares can be generated by cellular automata (CAs). A *cellular automaton* (CA) is a discrete dynamical system described by a regular lattice of cells, where each cell synchronously updates its value by applying a local rule to itself and its neighbouring cells. There has been considerable attention into the relationship between CAs and Latin squares, especially concerning CAs defined over finite fields [22–25]. In particular, the survey by Manzoni, Mariot and Menara [26] offers a comprehensive account of how combinatorial designs can be constructed via CAs, with a particular focus on mutually orthogonal Latin squares, and also reviews the applications of CA-based designs in areas such as cryptography and coding theory. Our study is motivated by the work of Mariot et al. [25], who found a construction of Latin squares generated by CAs.

The paper is organised as follows. Section 2 covers the necessary background on *F*-squares, cellular automata and prior research. Section 3 presents our main results. Finally, Section 4 summarises the key contributions of this study and explores promising directions for future research.

## 2 Background

In this section, we summarise some definitions, lemmas, and theorems that are used in our results. For further reading, we refer the reader to [9] and [25].

### 2.1 *F*-squares

The main objects of interest in this paper are *F*-squares.

**Definition 1** (Britz et al., [20]). *A* ***frequency square*** *or an* ***F-square****, of type* $\left(n;\lambda_{1},\lambda_{2},...,\lambda_{s}\right)$ *is an* n×n *array such that symbol i occurs* $\lambda_{i}$ *times in each row and* $\lambda_{i}$ *times in each column for each* i∈{1,...,s}*; necessarily* $\sum_{i=1}^{s}\lambda_{i}=n$.

The type $\left(n;\lambda_{1}^{2},\lambda_{3},\lambda_{4}^{2},...,\lambda_{s}\right)$ represents $\left(n;\lambda_{1},\lambda_{1},\lambda_{3},\lambda_{4},\lambda_{4},...,\lambda_{s}\right)$ while type (n;λ) represents (n;λ,λ,...,λ). That is, a *F*-square is of type (n;λ) if it contains $\frac{n}{\lambda }$ symbols, each of which appears λ times per row and λ per column. If λ=n, then we refer to the *F*-square as trivial, that is of type (*n*;*n*). A *F*-square of type (*n*; 1) is a Latin square of order *n*.

**Example 1.** *F-squares of type (6;2) and (5;2,1*^*3*^)


$$\begin{array}{cc} \begin{array}{cccccc} 1 & 2 & 3 & 3 & 2 & 1\\ 2 & 3 & 1 & 1 & 3 & 2\\ 3 & 1 & 2 & 2 & 1 & 3\\ 3 & 1 & 2 & 2 & 1 & 3\\ 2 & 3 & 1 & 1 & 3 & 2\\ 1 & 2 & 3 & 3 & 2 & 1 \end{array} & \;\begin{array}{ccccc} 1 & 2 & 3 & 4 & 1\\ 1 & 1 & 2 & 3 & 4\\ 4 & 1 & 1 & 2 & 3\\ 3 & 4 & 1 & 1 & 2\\ 2 & 3 & 4 & 1 & 1 \end{array} \end{array}$$

**Definition 2** (Diaconis and Gangolli, [27]). *Let* $𝐫=(r_{1},...,r_{m})$ *and* $𝐜=(c_{1},...,c_{n})$ *denote positive integer partitions of N. Let* $A_{rc}$ *denote the set of all* m×n *nonnegative integer matrices in which row i has sum* $r_{i}$ *and column j has sum* $c_{j}$*.*

When *m* = *n* and $r_{i}=c_{j}=k$, $A_{rc}$ becomes the set of n×n nonnegative integer matrices in which every row and every column sums to *k*. This class of matrices was first systematically studied by MacMahon in 1916 [27]. Notice that *F*-squares are members of this class. The row and column sum, *k*, of an *F*-square of type $\left(n;\lambda_{1},\lambda_{2},...,\lambda_{s}\right)$ is given by $\sum_{i=1}^{s}i\lambda_{i}$. When the *F*-square is a Latin square, *k* = *n*(*n* + 1)/2, and for a trivial *F*-square, *k* = *ns*, where *s* is the only element of the square.

**Definition 3** (Britz et al., [20]). *Mutually orthogonal F-squares (MOFS) are a set of F-squares which are pairwise orthogonal.*

### 2.2 Cellular automata

We are interested in one-dimensional CAs with no boundary conditions as finite compositions of functions, which defined as follows:

**Definition 4** (Mariot et al., [25]). *Let A be a finite alphabet of q elements and* n,d,q∈ℕ *with* n≥d*. The* ***No Boundary CA (NBCA)*** $ℱ:A^{n}\to A^{n-d+1}$ *of length n and diameter d determined by a local rule* $f:A^{d}\to A$ *is the vectorial function defined for all* $\bar{x}\in A^{n}$ *as*


$$\begin{array}{c} F(x_{1},x_{2},...,x_{n})=(f(x_{1},x_{2},...,x_{d}),f(x_{2},x_{3},...,x_{d+1}),...,f(x_{n-d+1},x_{n-d+2},...,x_{n})). \end{array}$$

When the finite alphabet is $A={𝔽}_{2}$, the local rule $f:{𝔽}_{2}^{d}\to {𝔽}_{2}$ can be represented by its truth table, and its decimal representation is the *Wolfram code* of the rule [24]. We use the notation CA to refer to one-dimensional CA with no boundary condition.

One widely studied class of CAs are known as *bipermutive CAs*. A CA with local rule *f* is *i*-permutive if, when all input variables except the *i*-th one are fixed, the function *f* becomes a bijection with respect to its *i*-th coordinate. The formal definition for an *i*-permutive is as follows:

**Definition 5** (Hudcová and Krásenský, [28]). *A CA* $ℱ:A^{n}\to A^{n-d+1}$ *induced by a local rule* $f:A^{d}\to A$ *is called* ***i-permutive*** *for* 1≤i≤d *if*


$$\begin{array}{c} f(x_{1},...,x_{i-1},\;˙\;,x_{i+1},...,x_{d}):A\to A, \end{array}$$

*is a bijection for all* $x_{1},...,x_{i-1},x_{i+1},...,x_{d}\in A$.

In the Boolean case $A={𝔽}_{2}$, an *i*-permutive local rule $f:{𝔽}_{2}^{d}\to {𝔽}_{2}$ can be written as:


$$\begin{array}{c} f(x_{1},x_{2},...,x_{d})=x_{i}+\varphi (x_{1},...,x_{i-1},x_{i+1},...,x_{d}), \end{array}$$

where $\varphi :{𝔽}_{2}^{d-1}\to {𝔽}_{2}$ is a function of d−1 variables, also called the *generating function* of *f* [29], and + corresponds to the sum operation over ${𝔽}_{2}$.

A CA is said to be *left permutive* if it is 1-permutive and *right permutive* if it is *d*-permutive. A CA which is both left and right permutive is said to be a *bipermutive* CA (BCA) [25].

For $A={𝔽}_{2}$, the local rule $f:{𝔽}_{2}^{d}\to {𝔽}_{q}$ is bipermutive if and only if there exists a (d−2)-variable function $\varphi :{𝔽}_{2}^{d-2}\to {𝔽}_{q}$ such that


$$\begin{array}{c} f(x_{1},x_{2},...,x_{d})=x_{1}+\varphi (x_{2},x_{3},...,x_{d-1})+x_{d}, \end{array}$$

for all $(x_{1},x_{2},...,x_{d})\in {𝔽}_{2}^{d}$ [25].

**Fig 1:**
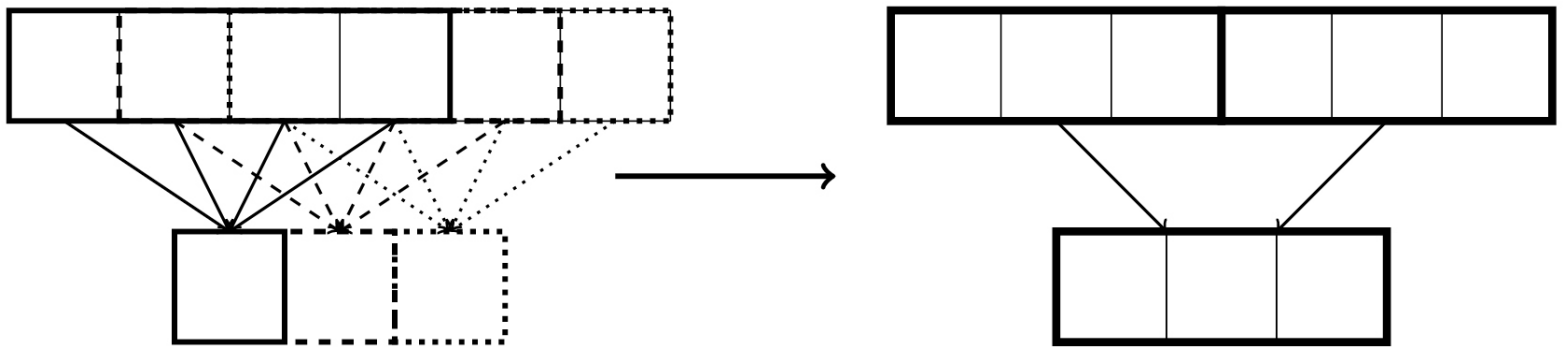
Block transformation of a CA. By blocking together d−1=3 sites, we can transform a CA of *d* = 4 into a CA with *d* = 2.

Note that a CA of any diameter *d* can be transformed into one with *d* = 2 by grouping sets of d−1 sites together as shown in Fig 1. Formally, this *block transformation* is an isomorphism, since it completely preserves the CA’s dynamics. Each new state is a function of exactly two prior states. We can then view a CA rule as a binary operation • on *A*,


$$\begin{array}{c} c=a•b. \end{array}$$

This approach was also taken in [30–33], which studied properties such as reversibility and permutivity, depending on what algebraic identities • satisfies.

By incorporating the construction of squares associated with CAs as described by Mariot et al. [25], together with the block transformation, we obtain the following definition for the square associated with a CA.

**Definition 6.** *Let A be an alphabet of q elements. The square associated to the CA* $ℱ:A^{2(d-1)}\to A^{d-1}$ *defined by the local rule* $f:A^{d}\to A$ *is the square matrix* $S_{ℱ}=[a_{ij}]$ *of order* $q^{d-1}\times q^{d-1}$ *with entries from* $\left\{1,2,...,q^{d-1}\right\}$ *defined for all* $1\leq i,j\leq q^{d-1}$ *as*


$$\begin{array}{c} a_{ij}={𝐱}_{i}•{𝐱}_{j}=ℱ({𝐱}_{i}||{𝐱}_{j}), \end{array}$$

*where* ${𝐱}_{i}||{𝐱}_{j}\in A^{2(d-1)}$ *denotes the concatenation of vectors* ${𝐱}_{i},{𝐱}_{j}\in A^{d-1}$.

**Fig 2:**
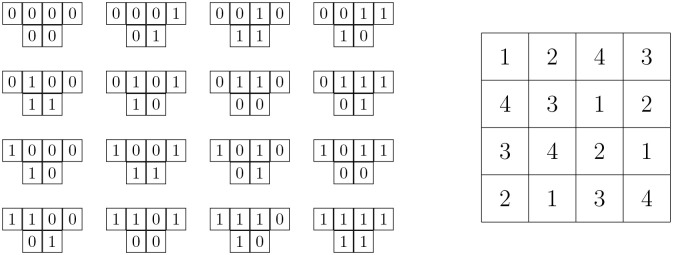
Truth table of ℱ defined by rule 150 and associated square $S_{F}$. Here, the entries in the table are obtained from the output of *f* as follows: 00↦1,01↦2,10↦3, and 11↦4.

Fig 2 depicts an example of square, $S_{F}$ of *d* = 3 over ${𝔽}_{2}$ (thus, the order $2^{3-1}=4)$ generated by the CA, $ℱ:F_{2}^{4}\to F_{2}^{2}$ which is defined by the local rule $f_{150}(x_{1},x_{2},x_{3})=x_{1}+x_{2}+x_{3}$.

The condition for generating Latin squares, that is, *F*-squares of type (*n*,1) from CAs is formalised in the following lemma:

**Lemma 1** (Mariot et al., [25]). *Let A be an alphabet of q symbols, and* d≥2*. Then, the square* $S_{ℱ}$ *of a bipermutive CA,* $ℱ:A^{2(d-1)}\to A^{d-1}$*, defined by the local rule* $f:A^{d}\to A$ *is a Latin square of order* $q^{d-1}$ *over* $X=\{1,...,q^{d-1}\}$*.*

A proof of the Lemma 1 based on Latin squares can be found in [25], and a similar proof using the characterisation of quasigroups is given in [32]. The necessary condition for constructing Latin squares from CAs, in the special case $A={𝔽}_{2}$, is discussed in a separate work (Manuscript titled “A new approach to construct frequency squares from cellular automata," submitted for publication by Joanne Hall, Kerri Morgan, Stella Stylianou, and Vindya Warnakulasooriyage, 2025). For any alphabets, a study based on block transformations is provided in another submitted work (Manuscript titled “Isotopy classes of Latin squares generated by cellular automata: an algebraic approach," submitted for publication by Joanne Hall, Kerri Morgan, Stella Stylianou, and Vindya Warnakulasooriyage, 2025).

In this study, we investigate whether CAs over ${𝔽}_{2}$ can generate *F*-squares other than Latin squares. In particular, we examine the row and column sums of the squares generated by the CA, which leads to the results presented in the following section.

## 3 Main results

Throughout, we assume that we have blocked together k=d−1 sites of the underlying CA, ℱ, with local rule is *f* of diameter *d* to produce a *d* = 2 blocked CA, whose local rule is a binary operation. The elements of ${𝔽}_{2}^{n}$ are always assumed to be lexicographically ordered unless stated otherwise. The elements of the square generated by the CA are illustrated in Table 1.

**Table 1:** Square ${𝒮}_{ℱ}$ generated from CA of diameter *d.*

•	${𝐱}_{1}$	${𝐱}_{2}$	⋯	${𝐱}_{j}$	⋯	${𝐱}_{2^{d-1}}$
${𝐱}_{1}$	$ℱ({𝐱}_{1}||{𝐱}_{1})$	$ℱ({𝐱}_{1}||{𝐱}_{2})$	⋯	$ℱ({𝐱}_{1}||{𝐱}_{j})$	⋯	$ℱ({𝐱}_{1}||{𝐱}_{2^{d-1}})$
${𝐱}_{2}$	$ℱ({𝐱}_{2}||{𝐱}_{1})$	$ℱ({𝐱}_{2}||{𝐱}_{2})$	⋯	$ℱ({𝐱}_{2}||{𝐱}_{j})$	⋯	$ℱ({𝐱}_{2}||{𝐱}_{2^{d-1}})$
⋮	⋮	⋮	⋱	⋮	⋱	⋮
${𝐱}_{i}$	$ℱ({𝐱}_{i}||{𝐱}_{1})$	$ℱ({𝐱}_{i}||{𝐱}_{2})$	⋯	$ℱ({𝐱}_{i}||{𝐱}_{j})$	⋯	$ℱ({𝐱}_{i}||{𝐱}_{2^{d-1}})$
⋮	⋮	⋮	⋱	⋮	⋱	⋮
${𝐱}_{2^{d-1}}$	$ℱ({𝐱}_{2^{d-1}}||{𝐱}_{1})$	$ℱ({𝐱}_{2^{d-1}}||{𝐱}_{2})$	⋯	$ℱ({𝐱}_{2^{d-1}}||{𝐱}_{j})$	⋯	$ℱ({𝐱}_{2^{d-1}}||{𝐱}_{2^{d-1}})$

The sum of any row *i* (denoted by $R_{i}$) and the sum of any column *j* (denoted by $C_{j}$) in the square generated by the CA are given as follows:


$$\begin{array}{ccc} R_{i} & = & \sum_{j=1}^{2^{d-1}}ℱ({\text{x}}_{i}||{\text{x}}_{j})\\ C_{j} & = & \sum_{i=1}^{2^{d-1}}ℱ({\text{x}}_{i}||{\text{x}}_{j}) \end{array}$$

Since the elements of ${𝔽}_{2}^{d-1}$ are arranged in lexicographically order, the (2m−1)th and the (2*m*)th elements differ only in their final bit while the (*m*)th and the $\left(m+2^{d-2}\right)$th elements differ only in their first bit for $m\in [1,2^{d-2}]$.

Now we consider the condition under which the column sums of columns (2m−1) and (2*m*), as well as the row sums of rows (*m*) and $\left(m+2^{d-2}\right)$, are equal. This restrictive case is important because, in an *F*-square from a CA, every column sums and row sums are equal; therefore, the column/row sum of any pair of columns/rows must also be equal.

Define, for $a,b\in {𝔽}_{2}$, the operation a+b:=a+b∈ℤ to denote integer (not modulo 2) addition. We extend this notation to sums of multiple ${𝔽}_{2}$-valued terms, where all additions are interpreted over ℤ.

**Lemma 2.** *Let* $ℱ:{𝔽}_{2}^{2(d-1)}\to {𝔽}_{2}^{d-1}$ *be a CA defined by a local rule* $f:{𝔽}_{2}^{d}\to {𝔽}_{2}$*. Then the column sums of the* (2m−1)*th and (2m)th columns in the square generated by a CA are equal if and only if*


$$\begin{array}{c} f(0,y_{1},...,y_{d-2},0)+f(1,y_{1},...,y_{d-2},0)=f(0,y_{1},...,y_{d-2},1)+f(1,y_{1},...,y_{d-2},1), \end{array}$$

*where* $(y_{1},y_{2},...,y_{d-2})\in {𝔽}_{2}^{d-2}$ *and* $m\in [1,2^{d-2}]$.

*Proof.* Consider two columns, (2m−1)th and (2*m*)th indexed by ${𝐱}_{2m-1}$ and ${𝐱}_{2m}$. Without loss of generality we may take ${𝐱}_{2m-1}=(y_{1},y_{2},...,y_{d-2},0)$ and ${𝐱}_{2m}=(y_{1},y_{2},...,y_{d-2},1)$, which differ only in their last bit. Let $C_{2m-1}$ and *C*_2*m*_ be the respective column sums:


$$\begin{array}{cc} C_{2m-1} & =\sum_{i=1}^{2^{d-1}}ℱ({\text{x}}_{i}||{\text{x}}_{2m-1})\\ & =\sum_{i=1}^{2^{d-1}}ℱ(x_{1,i},x_{2,i},...,x_{d-1,i}||y_{1},y_{2},...,y_{d-2},0)\\ & =\sum_{i=1}^{2^{d-1}}(f(x_{1,i},x_{2,i},...,y_{1}),f(x_{2,i},x_{3,i},...,y_{2}),...,f(x_{d-2,i},x_{d-1,i},y_{1},...,y_{d-2}),\\ & \;f(x_{d-1,i},y_{1},...,y_{d-2},0))\\ & =(\sum_{i=1}^{2^{d-1}}f(x_{1,i},x_{2,i},...,y_{1}),\sum_{i=1}^{2^{d-1}}f(x_{2,i},x_{3,i},...,y_{2}),...,\sum_{i=1}^{2^{d-1}}f(x_{d-1,i},y_{1},...,y_{d-2},0)) \end{array}$$

Similarly,


$$\begin{array}{cc} C_{2m} & =\sum_{i=1}^{2^{d-1}}ℱ({\text{x}}_{i}||{\text{x}}_{2m})\\ & =(\sum_{i=1}^{2^{d-1}}f(x_{1,i},x_{2,i},...,y_{1}),\sum_{i=1}^{2^{d-1}}f(x_{2,i},x_{3,i},...,y_{2}),...,\sum_{i=1}^{2^{d-1}}f(x_{d-1,i},y_{1},...,y_{d-2},1)) \end{array}$$

Note that all components of $C_{2m-1}$ and *C*_2*m*_ are identical except possibly the last, which depends on whether the final bit is 0 or 1. Now observe that for fixed $\left(y_{1},...,y_{d-2}\right)$, the final argument of *f* varies only in the first component $x_{d-1,i}$, which ranges over all $2^{d-1}$ binary vectors. In particular, $x_{d-1,i}\in \{0,1\}$, and both values appear exactly $2^{d-2}$ times among the ${𝐱}_{i}$. Therefore:

From $C_{2m-1}$:


$$\begin{array}{c} \sum_{i=1}^{2^{d-1}}f(x_{d-1,i},y_{1},...,y_{d-2},0)=2^{d-2}(f(0,y_{1},...,y_{d-2},0)+f(1,y_{1},...,y_{d-2},0)) \end{array}$$

From *C*_2*m*_:


$$\begin{array}{c} \sum_{i=1}^{2^{d-1}}f(x_{d-1,i},y_{1},...,y_{d-2},1)=2^{d-2}(f(0,y_{1},...,y_{d-2},1)+f(1,y_{1},...,y_{d-2},1)) \end{array}$$

Now, we conclude that *C*_2*m*+1_ and *C*_2*m*+2_ are equal if and only if:


$$\begin{array}{c} f(0,y_{1},...,y_{d-2},0)+f(1,y_{1},...,y_{d-2},0)=f(0,y_{1},...,y_{d-2},1)+f(1,y_{1},...,y_{d-2},1). \end{array}$$ (1)

We now examine the corresponding condition for row sums in the square generated by a CA. □

**Lemma 3.** *Let* $ℱ:{𝔽}_{2}^{2(d-1)}\to {𝔽}_{2}^{d-1}$ *be a CA defined by a local rule* $f:{𝔽}_{2}^{d}\to {𝔽}_{2}$*. Then the row sums of the (m)-th and* $\left(m+2^{d-2}\right)$*-th rows in the square generated by CA are equal if and only if*


$$\begin{array}{c} f(0,y_{2},...,y_{d-1},0)+f(0,y_{2},...,y_{d-1},1)=f(1,y_{2},...,y_{d-1},0)+f(1,y_{2},...,y_{d-1},1), \end{array}$$

*where* $(y_{2},y_{3},...,y_{d-1})\in {𝔽}_{2}^{d-2}$ *and* $m\in [1,2^{d-2}]$.

*Proof.* Consider two rows, (*m*)th and $\left(m+2^{d-2}\right)$th indexed by ${𝐱}_{m}$ and ${𝐱}_{m+2^{d-2}}$. Without loss of generality we may take ${𝐱}_{m}=(0,y_{2},...,y_{d-2},y_{d-1})$ and ${𝐱}_{m+2^{d-2}}=(1,y_{2},...,y_{d-2},y_{d-1})$, which differ only in their first bit. Let $R_{m}$ and $R_{m+2^{d-2}}$ be the respective row sums:


$$\begin{array}{cc} R_{m}= & \sum_{j=1}^{2^{d-1}}ℱ({\text{x}}_{m}||{\text{x}}_{j})\\ = & \sum_{j=1}^{2^{d-1}}ℱ(0,y_{2},...,y_{d-1}||x_{1,j},x_{2,j},...,x_{d-1,j})\\ = & \sum_{j=1}^{2^{d-1}}(f(0,y_{2},...,y_{d-1},x_{1,j}),f(y_{2},y_{3},...,x_{2,j}),...,f(y_{d-2},y_{d-1},x_{1,i},...,x_{d-2,j}),\\ & f(y_{d-1},x_{1,j},...,x_{d-1,j}))\\ = & \left(\sum_{j=1}^{2^{d-1}}f(0,y_{2},...,y_{d-1},x_{1,j}),\sum_{j=1}^{2^{d-1}}f(y_{2},y_{3},...,x_{2,j}),...,\sum_{j=1}^{2^{d-1}}f(y_{d-1},x_{1,j},...,x_{d-1,j})\right) \end{array}$$

Similarly,


$$\begin{array}{cc} R_{m+2^{d-2}} & =\sum_{j=1}^{2^{d-1}}ℱ({\text{x}}_{m+2^{d-2}}||{\text{x}}_{j})\\ & =(\sum_{j=1}^{2^{d-1}}f(1,y_{2},...,y_{d-1},x_{1,j}),\sum_{j=1}^{2^{d-1}}f(y_{2},y_{3},...,x_{2,j}),...,\sum_{j=1}^{2^{d-1}}f(y_{d-1},x_{1,j},...,x_{d-1,j})) \end{array}$$

Note that all components of $R_{m}$ and $R_{m+2^{d-2}}$ are identical except possibly the first, which depends on whether the first bit is 0 or 1. Now observe that for fixed $\left(y_{2},...,y_{d-1}\right)$, the final argument of *f* varies only in the last component *x*_1,*j*_, which ranges over all $2^{d-1}$ binary vectors. In particular, $x_{1,j}\in \{0,1\}$, and both values appear exactly $2^{d-2}$ times among the ${𝐱}_{j}$. Therefore:

From $R_{m}$:


$$\begin{array}{c} \sum_{j=1}^{2^{d-1}}f(0,y_{2},...,y_{d-1},x_{1,j})=2^{d-2}(f(0,y_{2},...,y_{d-1},0)+f(0,y_{2},...,y_{d-1},1)) \end{array}$$

From $R_{m+2^{d-2}}$:


$$\begin{array}{c} \sum_{j=1}^{2^{d-1}}f(1,y_{2},...,y_{d-1},x_{1,j})=2^{d-2}(f(1,y_{2},...,y_{d-1},0)+f(1,y_{2},...,y_{d-1},1)) \end{array}$$

Now, we conclude that $R_{m}$ and $R_{m+2^{d-2}}$ are equal if and only if:


$$\begin{array}{c} f(0,y_{2},...,y_{d-1},0)+f(0,y_{2},...,y_{d-1},1)=f(1,y_{2},...,y_{d-1},0)+f(1,y_{2},...,y_{d-1},1). \end{array}$$ (2)

□

From this point forward, we will refer to pair of columns that differ only in their final (rightmost) bit, for example, the (2m−1)st and (2*m*)nd columns, as *paired columns*. Similarly, we refer to pair of rows that differ only in their first (leftmost) bit, such as the (*m*)th and $\left(m+2^{d-2}\right)$th rows, as *paired rows*.

We have established two conditions under which paired columns sums and paired rows sums in a square generated by a CA are equal. We now turn our attention to identifying the local rules of CAs over ${𝔽}_{2}$ that satisfy those conditions.

**Lemma 4.** *Let* $ℱ:{𝔽}_{2}^{2(d-1)}\to {𝔽}_{2}^{d-1}$ *be a CA defined by a local rule* $f:{𝔽}_{2}^{d}\to {𝔽}_{2}$*. Then, the column sums of paired columns in the square generated by CA are equal if and only if the local rule f is of the form:*


$$\begin{array}{c} f(x_{1},x_{2},...,x_{d})=g_{1}(x_{2},x_{3},...,x_{d-1})+x_{1}h_{1}(x_{2},x_{3},...,x_{d-1}), \end{array}$$

*or*


$$\begin{array}{c} f(x_{1},x_{2},...,x_{d})=g_{1}(x_{2},x_{3},...,x_{d-1})+(x_{1}+x_{d})h_{1}(x_{2},x_{3},...,x_{d-1}), \end{array}$$

*for some functions* $g_{1},h_{1}:{𝔽}_{2}^{d-2}\to {𝔽}_{2}$.

*Proof.* Any Boolean function can be expressed by fixing a variable and decomposing the function in terms of the remaining variables. In particular, by fixing $x_{d}$, the local rule *f* can be written as:


$$\begin{array}{c} f(x_{1},x_{2},...,x_{d})=f_{1}(x_{1},x_{2},...,x_{d-1})+x_{d}f_{2}(x_{1},x_{2},...,x_{d-1}), \end{array}$$ (3)

for some functions $f_{1},f_{2}:{𝔽}_{2}^{d-1}\to {𝔽}_{2}$.

We can further decompose each $f_{i}$ for *i* = 1,2 by fixing *x*_1_ and expressing it in terms of functions of d−2 variables. This yields:


$$\begin{array}{cc} f(x_{1},x_{2},...,x_{d})= & \;g_{1}(x_{2},x_{3},...,x_{d-1})+x_{1}h_{1}(x_{2},x_{3},...,x_{d-1})\\ & +x_{d}g_{2}(x_{2},x_{3},...,x_{d-1})+x_{1}x_{d}h_{2}(x_{2},x_{3},...,x_{d-1}), \end{array}$$ (4)

for some functions $g_{1},g_{2},h_{1},h_{2}:{𝔽}_{2}^{d-2}\to {𝔽}_{2}$. To determine the column sum of paired columns are equal, we consider the condition in Eq (1) of Lemma 2, which involves evaluating the local rule at four points. Take any $y_{1},...,y_{d-2}\in {𝔽}_{2}$. Using the decomposition in Eq (4), we evaluate:


$$\begin{array}{c} f(0,y_{1},...,y_{d-2},0)=g_{1}(y_{1},...,y_{d-2}) \end{array}$$ (5)

$$\begin{array}{c} f(1,y_{1},...,y_{d-2},0)=g_{1}(y_{1},...,y_{d-2})+h_{1}(y_{1},...,y_{d-2}) \end{array}$$ (6)

$$\begin{array}{c} f(0,y_{1},...,y_{d-2},1)=g_{1}(y_{1},...,y_{d-2})+g_{2}(y_{1},...,y_{d-2}) \end{array}$$ (7)

$$\begin{array}{c} \begin{array}{cc} f(1,y_{1},...,y_{d-2},1)=g_{1}(y_{1},...,y_{d-2})+g_{2}(y_{1},...,y_{d-2}) & +h_{1}(y_{1},...,y_{d-2})\\ & +h_{2}(y_{1},...,y_{d-2}) \end{array} \end{array}$$ (8)

From Eq (1) and the evaluations in (5)–(8), we derive:


$$\begin{array}{ccc} \overset{T_{1}}{\overbrace{f^{\left(d\right)}(0,y_{1},...,y_{d-2},0)}}+\overset{T_{2}}{\overbrace{f^{\left(d\right)}(1,y_{1},...,y_{d-2},0)}} & = & \left(g_{1})+(g_{1}+h_{1}\right) \end{array}$$ (9)

$$\begin{array}{ccc} \underset{T_{3}}{\underbrace{f^{\left(d\right)}(0,y_{1},...,y_{d-2},1)}}+\underset{T_{4}}{\underbrace{f^{\left(d\right)}(1,y_{1},...,y_{d-2},1)}} & = & \left(g_{1}+g_{2})+(g_{1}+g_{2}+h_{1}+h_{2}\right) \end{array}$$ (10)

All functions $g_{i}$ and $h_{i}$ are evaluated at $\left(y_{1},...,y_{d-2}\right)$. Note that each term in Eq (1) is an element of ${𝔽}_{2}$, i.e., either 0 or 1. For the function *f* to yield equal column sums, Eq (9) and Eq (10) must evaluate to the same value. We proceed by analysing two cases based on this value according to the Table 2.

**Table 2:** Value table corresponds to $T_{1},T_{2},T_{3}$ and *T*_4_.

*T*_1_	*T*_2_	*T*_3_	*T*_4_	$T_{1}\mathrm{\backslash pmb}+T_{2}$	$T_{3}\mathrm{\backslash pmb}+T_{4}$
0	0	0	0	0	0
0	1	0	0	1	0
1	0	0	0	1	0
1	1	0	0	2	0
0	0	0	1	0	1
0	1	0	1	1	1
1	0	0	1	1	1
1	1	0	1	2	1
0	0	1	0	0	1
0	1	1	0	1	1
1	0	1	0	1	1
1	1	1	0	2	1
0	0	1	1	0	2
0	1	1	1	1	2
1	0	1	1	1	2
1	1	1	1	2	2

If $T_{1}+T_{2}=T_{3}+T_{4}$, where $T_{1},T_{2},T_{3},T_{4}\in {𝔽}_{2}$, then the pair $\left(T_{1},T_{2}\right)$ and $\left(T_{3},T_{4}\right)$ must contain the same multiset of values. This occurs in exactly two cases:

- **Case I:** $T_{1}=T_{3}$ and $T_{2}=T_{4}$
- **Case II:** $T_{1}=T_{4}$ and $T_{2}=T_{3}$

Note that the two cases overlap precisely when $T_{1}=T_{2}=T_{3}=T_{4}$. That is, both cases apply when all four variables are equal – either all 0 or all 1.

**Case I:**

We are given the following congruences modulo 2:


$$\begin{array}{cc} g_{1} & \equiv g_{1}+g_{2}\;(\mathrm{mod}\;2) \end{array}$$ (11)

$$\begin{array}{cc} g_{1}+h_{1} & \equiv g_{1}+g_{2}+h_{1}+h_{2}\;(\mathrm{mod}\;2) \end{array}$$ (12)

From Eq (11):


$$\begin{array}{c} g_{2}\equiv 0\;\Rightarrow \;g_{2}=0\; \end{array}$$

Substituting *g*_2_ = 0 into Eq (12), we obtain


$$\begin{array}{c} g_{1}+h_{1}\equiv g_{1}+h_{1}+h_{2}\;\Rightarrow \;h_{2}\equiv 0\;\Rightarrow \;h_{2}=0\; \end{array}$$

Therefore, both conditions are satisfied if and only if:


$$\begin{array}{c} g_{2}=0,\;h_{2}=0 \end{array}$$

Now, since $\left(y_{1},...,y_{d-2}\right)$ range over all values in ${𝔽}_{2}^{d-2}$, the equations above hold for all such values. Hence, substituting these into Eq (4), we obtain:


$$\begin{array}{c} f(x_{1},...,x_{d})=g_{1}(x_{2},x_{3},...,x_{d-1})+x_{1}h_{1}(x_{2},x_{3},...,x_{d-1}). \end{array}$$

**Case II:**

$$\begin{array}{cc} g_{1} & \equiv g_{1}+g_{2}+h_{1}+h_{2}\;(\mathrm{mod}\;2) \end{array}$$ (13)

$$\begin{array}{cc} g_{1}+h_{1} & \equiv g_{1}+g_{2}\;(\mathrm{mod}\;2) \end{array}$$ (14)

From Eq (14):


$$\begin{array}{c} g_{2}\equiv h_{1}\;(\mathrm{mod}\;2) \end{array}$$ (15)

From Eq (13) and Eq (15):


$$\begin{array}{c} g_{2}+h_{1}+h_{2}\equiv 0\;\Rightarrow \;h_{2}\equiv 0\Rightarrow \;h_{2}=0 \end{array}$$

Now, since $\left(y_{1},...,y_{d-2}\right)$ range over all values in ${𝔽}_{2}^{d-2}$, the equations above hold for all such values. Hence, substituting these into Eq (4), we get:


$$\begin{array}{cc} f(x_{1},x_{2},...,x_{d}) & =g_{1}(x_{2},x_{3},...,x_{d-1})+x_{1}h_{1}(x_{2},x_{3}...,x_{d-1})+x_{d}h_{1}(x_{2},x_{3},...,x_{d-1})\\ & =g_{1}(x_{2},x_{3},...,x_{d-1})+(x_{1}+x_{d})h_{1}(x_{2},x_{3},...,x_{d-1}) \end{array}$$

□

**Lemma 5.** *Let* $ℱ:{𝔽}_{2}^{2(d-1)}\to {𝔽}_{2}^{d-1}$ *be a CA defined by a local rule* $f:{𝔽}_{2}^{d}\to {𝔽}_{2}$*. Then, the row sums of paired rows in the square generated by CA are equal if and only if the local rule f is of the form:*


$$\begin{array}{c} f(x_{1},x_{2},...,x_{d})=g_{1}(x_{2},x_{3},...,x_{d-1})+x_{d}g_{2}(x_{2},x_{3},...,x_{d-1}), \end{array}$$

*or*


$$\begin{array}{c} f(x_{1},x_{2},...,x_{d})=g_{1}(x_{2},x_{3},...,x_{d-1})+(x_{1}+x_{d})g_{2}(x_{2},x_{3},...,x_{d-1}), \end{array}$$

*for some functions* $g_{1},g_{2}:{𝔽}_{2}^{d-2}\to {𝔽}_{2}$.

*Proof.* Similar to the proof of Lemma 4, to determine when paired row sums are equal, we consider the row sum condition (see Eq (2)), which involves evaluating the local rule at four points. Take any $y_{2},...,y_{d-1}\in {𝔽}_{2}$. Using the decomposition in Eq (4), we evaluate:


$$\begin{array}{ccc} f(0,y_{2},...,y_{d-1},0) & = & g_{1}(y_{2},...,y_{d-1}) \end{array}$$ (16)

$$\begin{array}{ccc} f(0,y_{2},...,y_{d-1},1) & = & g_{1}(y_{2},...,y_{d-1})+g_{2}(y_{2},...,y_{d-1}) \end{array}$$ (17)

$$\begin{array}{ccc} f(1,y_{2},...,y_{d-1},0) & = & g_{1}(y_{2},...,y_{d-1})+h_{1}(y_{2},...,y_{d-1}) \end{array}$$ (18)

$$\begin{array}{cc} f(1,y_{2},...,y_{d-1},1) & =g_{1}(y_{2},...,y_{d-1})+g_{2}(y_{2},...,y_{d-1})+h_{1}(y_{2},...,y_{d-1})\\ & \;+h_{2}(y_{2},...,y_{d-1}) \end{array}$$ (19)

Similarly, from Eq (2) and the evaluations in (16)–(19), we derive:


$$\begin{array}{ccc} \overset{T_{5}}{\overbrace{f^{\left(d\right)}(0,y_{2},...,y_{d-1},0)}}+\overset{T_{6}}{\overbrace{f^{\left(d\right)}(0,y_{2},...,y_{d-1},1)}} & = & \left(g_{1})+(g_{1}+g_{2}\right) \end{array}$$ (20)

$$\begin{array}{ccc} \underset{T_{7}}{\underbrace{f^{\left(d\right)}(1,y_{2},...,y_{d-1},0)}}+\underset{T_{8}}{\underbrace{f^{\left(d\right)}(1,y_{2},...,y_{d-1},1)}} & = & \left(g_{1}+h_{1})+(g_{1}+g_{2}+h_{1}+h_{2}\right) \end{array}$$ (21)

All functions $g_{i}$ and $h_{i}$ are evaluated at $\left(y_{2},...,y_{d-1}\right)$. Note that each term in Eq (2) is an element of ${𝔽}_{2}$, i.e., either 0 or 1. For the function *f* to yield equal row sums, Eq (20) and Eq (21) must evaluate to the same value. We proceed by analysing two cases based on this value.

If $T_{5}+T_{6}=T_{7}+T_{8}$, where $T_{5},T_{6},T_{7},T_{8}\in {𝔽}_{2}$, then the pair $\left(T_{5},T_{6}\right)$ and $\left(T_{7},T_{8}\right)$ must contain the same multiset of values. This occurs in exactly two cases:

- **Case I:** $T_{5}=T_{7}$ and $T_{6}=T_{7}$
- **Case II:** $T_{5}=T_{8}$ and $T_{6}=T_{7}$

Note that the two cases overlap precisely when $T_{5}=T_{6}=T_{7}=T_{8}$. That is, both cases apply when all four variables are equal – either all 0 or all 1.

**Case I:**

We are given the following congruences modulo 2:


$$\begin{array}{cc} g_{1} & \equiv g_{1}+h_{1}\;(\mathrm{mod}\;2) \end{array}$$ (22)

$$\begin{array}{cc} g_{1}+g_{2} & \equiv g_{1}+g_{2}+h_{1}+h_{2}\;(\mathrm{mod}\;2) \end{array}$$ (23)

From Eq (22):


$$\begin{array}{c} h_{1}\equiv 0\;\Rightarrow \;h_{1}=0 \end{array}$$

Substituting *h*_1_ = 0 into Eq (23), we obtain


$$\begin{array}{c} g_{1}+g_{2}\equiv g_{1}+g_{2}+h_{2}\;\Rightarrow \;h_{2}\equiv 0\;\Rightarrow \;h_{2}=0 \end{array}$$

Therefore, both conditions are satisfied if and only if:


$$\begin{array}{c} h_{1}=0,\;h_{2}=0 \end{array}$$

Now, since $\left(y_{2},...,y_{d-1}\right)$ range over all values in ${𝔽}_{2}^{d-2}$, and the equations above hold for all such values. Hence, substituting these into Eq (4), we obtain:


$$\begin{array}{c} f(x_{1},x_{2},...,x_{d})=g_{1}(x_{2},x_{3},...,x_{d-1})+x_{d}g_{2}(x_{2},x_{3},...,x_{d-1}). \end{array}$$

**Case II:**

We are now given the following congruences modulo 2:


$$\begin{array}{cc} g_{1} & \equiv g_{1}+g_{2}+h_{1}+h_{2}\;(\mathrm{mod}\;2) \end{array}$$ (24)

$$\begin{array}{cc} g_{1}+g_{2} & \equiv g_{1}+h_{1}\;(\mathrm{mod}\;2) \end{array}$$ (25)

From Eq (25):


$$\begin{array}{c} g_{2}\equiv h_{1}\;(\mathrm{mod}\;2) \end{array}$$ (26)

From Eq (24) and Eq (26):


$$\begin{array}{c} g_{2}+h_{1}+h_{2}\equiv 0\;\Rightarrow \;h_{2}\equiv 0\;\Rightarrow \;h_{2}=0\; \end{array}$$

Now, since $\left(y_{2},...,y_{d-1}\right)$ range over all values in ${𝔽}_{2}^{d-2}$, and the equations above hold for all such values. Hence, substituting these into Eq (4), we get:

$\begin{array}{cc} f(x_{1},x_{2},...,x_{d}) & =g_{1}(x_{2},x_{3},...,x_{d-1})+x_{1}g_{2}(x_{2},x_{3},...,x_{d-1})+x_{d}g_{2}(x_{2},x_{3},...,x_{d-1})\\ & =g_{1}(x_{2},x_{3},...,x_{d-1})+(x_{1}+x_{d})g_{2}(x_{2},x_{3},...,x_{d-1}) \end{array}$

□

We now examine the identification of local rules that simultaneously yield paired rows with equal row sums and paired columns with equal column sums.

**Lemma 6.** *Let* $ℱ:{𝔽}_{2}^{2(d-1)}\to {𝔽}_{2}^{d-1}$ *be a CA defined by a local rule* $f:{𝔽}_{2}^{d}\to {𝔽}_{2}$*. Then, the column sums of paired columns and the row sums of paired rows in the square generated by CA are equal simultaneously if and only if the local rule f is of one of the following forms:*


$$\begin{array}{c} f(x_{1},x_{2},...,x_{d})=g_{1}(x_{2},x_{3},...,x_{d-1}), \end{array}$$

*or*


$$\begin{array}{c} f(x_{1},x_{2},...,x_{d})=g_{1}(x_{2},x_{3},...,x_{d-1})+(x_{1}+x_{d})g_{2}(x_{2},x_{3},...,x_{d-1}), \end{array}$$

*where* $g_{1},g_{2}:{𝔽}_{2}^{d-2}\to {𝔽}_{2}$.

*Proof.* From Lemma 4, the column sums of paired columns in the square generated by ℱ are equal if and only if the local rule *f* is of the form


$$\begin{array}{c} f=\left\{ \begin{array}{c} g_{1}(x_{2},...,x_{d-1})+x_{1}\;h_{1}(x_{2},...,x_{d-1})\\ g_{1}(x_{2},...,x_{d-1})+(x_{1}+x_{d})\;h_{1}(x_{2},...,x_{d-1}) \end{array}\right. \end{array}$$

From Lemma 5, the row sums of paired rows are equal if and only if


$$\begin{array}{c} f=\left\{ \begin{array}{c} g_{1}(x_{2},...,x_{d-1})+x_{d}\;g_{2}(x_{2},...,x_{d-1})\\ g_{1}(x_{2},...,x_{d-1})+(x_{1}+x_{d})\;g_{2}(x_{2},...,x_{d-1}) \end{array}\right. \end{array}$$

The common form of the local rule for both Lemma 4 and Lemma 5 is:


$$\begin{array}{c} f(x_{1},x_{2},...,x_{d})=g_{1}(x_{2},...,x_{d-1})+(x_{1}+x_{d})g_{2}(x_{2},...,x_{d-1}), \end{array}$$

which simultaneously satisfies both conditions and represents one possible form of the local rule functions.

Now, consider the case where $f(x_{1},...,x_{d})=g_{1}(x_{2},...,x_{d-1})$. This corresponds to choosing $h_{1}\equiv 0$ in Lemma 4 and $g_{2}\equiv 0$ in Lemma 5, so that


$$\begin{array}{ccc} f & = & g_{1}(x_{2},...,x_{d-1})+x_{1}\cdot 0=g_{1}(x_{2},...,x_{d-1}),\\ f & = & g_{1}(x_{2},...,x_{d-1})+x_{d}\cdot 0=g_{1}(x_{2},...,x_{d-1}). \end{array}$$

Thus, the function $f(x_{1},x_{2},...,x_{d})=g_{1}(x_{2},x_{3},...,x_{d-1})$ satisfies the structural conditions in both lemmas and represents the second possible form of the local rule functions. □

Lemma 6 establishes that for squares generated by CAs, the column sums of any paired columns and the row sums of any paired rows are equal if the local rule *f* takes one of the following forms:


$$\begin{array}{c} f=\left\{ \begin{array}{cc} g_{1}(x_{2},x_{3},...,x_{d-1}) & \text{(Type 1)}\\ g_{1}(x_{2},x_{3},...,x_{d-1})+(x_{1}+x_{d})\;g_{2}(x_{2},x_{3},...,x_{d-1}) & \text{(Type 2)} \end{array}\right. \end{array}$$ (27)

For convenience, let us denote the two local rule functions as Type 1 and Type 2, respectively.

Since a necessary condition for a square to be a *F*-square is that all row and column sums are equal, it is natural to ask whether such local rules always generate *F*-squares. To investigate this, we present two counterexamples for each type when *d* = 3, demonstrating that the local rules do not, in general, produce *F*-squares.

**Fig 3:**
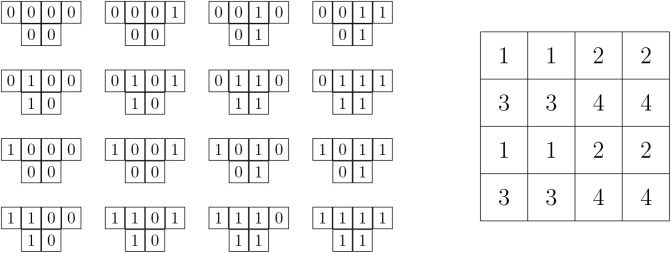
Truth table of ${ℱ}^{\left(a\right)}$ and the associated square ${S_{F}}^{\left(a\right)}$. Shown in the square ${S_{F}}^{\left(a\right)}$ for *d* = 3 over ${𝔽}_{2}$, generated by the CA ${ℱ}^{\left(a\right)}:F_{2}^{4}\to F_{2}^{2}$ with the local rule $f^{\left(a\right)}(x_{1},x_{2},x_{3})=x_{2}$.

**Fig 4:**
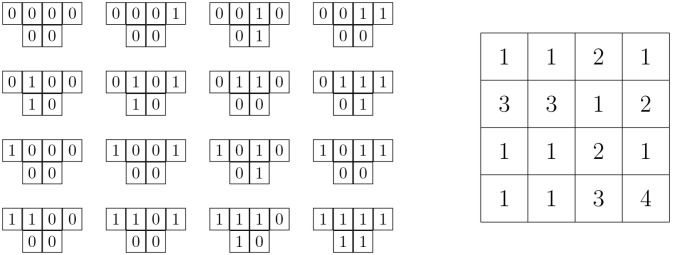
Truth table of ${ℱ}^{\left(b\right)}$ and the associated square ${S_{F}}^{\left(b\right)}$. Shown in the square ${S_{F}}^{\left(b\right)}$ for *d* = 3 over ${𝔽}_{2}$, generated by the CA ${ℱ}^{\left(b\right)}:F_{2}^{4}\to F_{2}^{2}$ with the local rule $f^{\left(b\right)}(x_{1},x_{2},x_{3})=x_{2}+x_{2}(x_{1}+x_{3})$.

Two squares ${S_{F}}^{\left(a\right)}$ and ${S_{F}}^{\left(b\right)}$, shown in Figs 3 and 4, respectively, are not *F*-squares. More importantly, we observe that in ${S_{F}}^{\left(a\right)}$, the paired columns and paired rows are identical. This occurs because the first case, *g*_1_, is independent of the leftmost and rightmost variables of *f*. However, the sums of non-paired columns and rows differ, indicating that uniformity in sums is not guaranteed beyond paired positions. This suggests a need to investi*g*ate further which form of Type 1 local rules produce squares with equal column and equal row sum.

**Proposition 1.** *Let* $ℱ:{𝔽}_{2}^{2(d-1)}\to {𝔽}_{2}^{d-1}$ *be a CA defined by the local rule* $f:{𝔽}_{2}^{d}\to {𝔽}_{2}$ *given by*


$$\begin{array}{c} f(x_{1},x_{2},...,x_{d})=g_{1}(x_{2},x_{3},...,x_{d-1}). \end{array}$$

*Then the column sums of every pair of two columns and the row sums of every pair of two rows in the square generated by CA are equal if and only if*


$$\begin{array}{c} g_{1}(x_{2},x_{3},...,x_{d-1})=A_{0}, \end{array}$$

*for some constant* $A_{0}\in {𝔽}_{2}$.

*Proof.* First, we consider the column sum requirement. Let two distinct columns be indexed by ${𝐱}_{s}=(y_{1},y_{2},...,y_{d-2},y_{d-1})$ and ${𝐱}_{t}=(z_{1},z_{2},...,z_{d-2},z_{d-1})$, where $(y_{1},...,y_{d-2},y_{d-1})\text{≠}(z_{1},...,z_{d-2},z_{d-1})\in {𝔽}_{2}^{d-1}$. The column sums are then be given by:


$$\begin{array}{ccc} C_{s} & = & \sum_{i=1}^{2^{d-1}}ℱ({\text{x}}_{i}||{\text{x}}_{s})\\ C_{t} & = & \sum_{i=1}^{2^{d-1}}ℱ({\text{x}}_{i}||{\text{x}}_{t}) \end{array}$$

Without loss of generality, we may assume that all components of $C_{s}$ and $C_{t}$ are identical except possibly the last, namely $C_{s,d}$ and $C_{t,d}$:


$$\begin{array}{ccc} C_{s,d} & = & f(0,y_{1},...,y_{d-2},y_{d-1})+f(1,y_{1},...,y_{d-2},y_{d-1})=g_{1}(y_{1},...,y_{d-2})+g_{1}(y_{1},...,y_{d-2}),\\ C_{t,d} & = & f(0,z_{1},...,z_{d-2},z_{d-1})+f(1,z_{1},...,z_{d-2},z_{d-1})=g_{1}(z_{1},...,z_{d-2})+g_{1}(z_{1},...,z_{d-2}). \end{array}$$

This calculation yields equal only if $g_{1}(y_{1},...,y_{d-2})=g_{1}(z_{1},...,z_{d-2})$ for all inputs, which implies that *g*_1_ is constant:


$$\begin{array}{c} g_{1}(x_{2},x_{3},...,x_{d-1})=A_{0},\;\text{where }A_{0}\in {𝔽}_{2}. \end{array}$$

In the similar way, we can work on the row sum requirement as well. Thus, we conclude that


$$\begin{array}{c} f(x_{1},x_{2},...,x_{d})=g_{1}(x_{2},x_{3},...,x_{d-1})=A_{0}. \end{array}$$

Moreover, if $g_{1}(x_{2},x_{3},...,x_{d-1})=A_{0}$ is constant, then the output of the CA is uniform, and every entry in the resulting square is the same. Therefore, every row and every column has the same sum. This completes the proof. □

In contrast, in ${S_{F}}^{\left(b\right)}$, although column sums of any paired columns and the row sums of any paired rows are equal, the entries themselves differ. For instance, the entries in the third column are {2,1,2,3}, while those in the forth column are {1,2,1,4}.

In addition to having equal sums for paired columns and paired rows, it is also necessary that the paired columns and paired rows contain the same elements. The local rules of Type 2 arise from the second cases of Lemma 4 and Lemma 5. Therefore, an additional condition must be imposed in the second case to ensure that paired rows and paired columns have the same multiset of elements.

**Proposition 2.** *Let* $ℱ:{𝔽}_{2}^{2(d-1)}\to {𝔽}_{2}^{d-1}$ *be a CA defined by the local rule* $f:{𝔽}_{2}^{d}\to {𝔽}_{2}$ *given by*


$$\begin{array}{c} f(x_{1},x_{2},...,x_{d})=g_{1}(x_{2},x_{3},...,x_{d-1})+(x_{1}+x_{d})g_{2}(x_{2},x_{3},...,x_{d-1}). \end{array}$$

*Then, the column sums of paired columns containing the same multiset of elements and the row sums of paired rows containing the same multiset of elements in the square generated by CA are equal simultaneously if and only if*


$$\begin{array}{c} g_{2}(x_{2},x_{3},...,x_{d-1})=1. \end{array}$$

*Proof.* First consider Case II of Lemma 4, where the condition is $T_{1}=T_{4}$ and $T_{2}=T_{3}$. Here we have the additional requirement that $T_{1}\text{≠}T_{2}$ or $T_{3}\text{≠}T_{4}$. This is necessary because, as illustrated in Fig 4, we observe that in some paired columns, $T_{1}=T_{4}=T_{2}=T_{3}$, while the other paired columns, $T_{1}=T_{4}$ and $T_{2}=T_{3}$ with $T_{1}\text{≠}T_{2}$, which disturbs consistency across the columns. Therefore, we require $T_{1}\text{≠}T_{2}$ or $T_{3}\text{≠}T_{4}$ to maintain consistency. Note that we do not consider the case $T_{1}=T_{4}=T_{2}=T_{3}$ here, as it overlaps with Type 1. Without loss of generality, we may assume $T_{2}=T_{1}+1$. Thus, we have:


$$\begin{array}{cc} g_{1} & \equiv g_{1}+g_{2}+h_{1}+h_{2}\;(\mathrm{mod}\;2) \end{array}$$ (28)

$$\begin{array}{cc} g_{1}+h_{1} & \equiv g_{1}+g_{2}\;(\mathrm{mod}\;2) \end{array}$$ (29)

$$\begin{array}{cc} g_{1}+1 & \equiv g_{1}+h_{1}\;(\mathrm{mod}\;2) \end{array}$$ (30)

From Eq (29):


$$\begin{array}{c} g_{2}\equiv h_{1}\;(\mathrm{mod}\;2) \end{array}$$ (31)

From Eq (28) and Eq (31):


$$\begin{array}{c} g_{2}+h_{1}+h_{2}\equiv 0\;\Rightarrow \;h_{2}=0 \end{array}$$

From Eq (30):


$$\begin{array}{c} h_{1}=1 \end{array}$$

Now, since $\left(y_{1},...,y_{d-2}\right)$ range over all values in ${𝔽}_{2}^{d-2}$, the equations above hold for all such values. Hence, substituting these into Eq (4), we get:


$$\begin{array}{cc} f(x_{1},x_{2},...,x_{d}) & =g_{1}(x_{2},x_{3},...,x_{d-1})+x_{1}h_{1}(x_{2},x_{3}...,x_{d-1})+x_{d}h_{1}(x_{2},x_{3},...,x_{d-1})\\ & =g_{1}(x_{2},x_{3},...,x_{d-1})+(x_{1}+x_{d})h_{1}(x_{2},x_{3},...,x_{d-1})\\ & =g_{1}(x_{2},x_{3},...,x_{d-1})+x_{1}+x_{d} \end{array}$$

We can achieve the same result by considering Case II of Lemma 5.□

Now, let us consider the local rule functions identified previously. From Proposition 1, we have the local rule function $f(x_{1},x_{2},...,x_{d})=A_{0}$, where $A_{0}\in {𝔽}_{2}$. From from Proposition 2, the local rule function is $f(x_{1},x_{2},...,x_{d})=x_{1}+g_{1}(x_{2},x_{3},...,x_{d-1})+x_{d}$, which represents bipermutive local rules. Thus, we have found that equal row and column sums are achieved when the local rule is either a constant function or bipermutive. When the local rule is $f(x_{1},x_{2},...,x_{d})=A_{0}$, all entries of the square generated by CA are the same, resulting in a trivial *F*-square. When the local rule is bipermutive, the square is a Latin square. Therefore, we arrive at the following main result:

**Theorem 1.** *Let* $ℱ:{𝔽}_{2}^{2(d-1)}\to {𝔽}_{2}^{d-1}$ *be a CA defined by a local rule* $f:{𝔽}_{2}^{d}\to {𝔽}_{2}$*. Then, the F-square generated by CA is either:*

- *a trivial square (type* $\left(2^{d-1};2^{d-1}\right)$*), or*
- *a Latin square (type* $\left(2^{d-1};1\right)$*).*

This result highlights a specific limitation of the binary setting. The entries of the CA-generated square are symbols drawn from an alphabet of size $2^{d-1}$, obtained by encoding vectors over ${𝔽}_{2}$, so that each coordinate of a symbol takes the value 0 or 1. The *F*-square condition requires that each symbol occurs with the same total frequency in every row and every column. Over ${𝔽}_{2}$, the equal‑frequency requirement, together with the constraints imposed by the underlying local rule, leads to a highly restricted class of behaviours: either the symbols are distributed uniformly across all rows and columns, so that the resulting array is a Latin square, or the square is trivial, with each row and column taking a constant value. In other words, there are no “intermediate” frequency patterns arising from binary CAs in this setting.

## 4 Conclusion

In this paper, we investigated the existence of *F*-squares generated by CAs over ${𝔽}_{2}$. Our primary approach was to analyse the row sums and column sums of the square produced by the CA, noting that these sums are equal in a *F*-square. We directed our attention to specific pairs of columns and rows, namely, pair of columns whose binary representation differ only in their final (rightmost) bit (for example, the (2m−1)th and (2*m*)th columns), refereed to as paired columns, and pair of rows whose binary representation differ only in their first (leftmost) bit (such as the (*m*)th and $\left(m+2^{d-2}\right)$th rows), refereed to as paired rows. These pairs were selected because they provide a focused and tractable basis for analysis. Moreover, ensuring that all such pairs have equal sums is a necessary condition for constructing an *F*-square from a CA.

We established two conditions under which paired columns sums and paired rows sums in a square generated by a CA are equal. We then focused on identifying the local rules of CAs over ${𝔽}_{2}$ that satisfy these conditions. In particular, we examined the local rules that simultaneously yield paired columns with equal column sums and paired rows with equal row sums, and identified two types: Type 1, defined by $f(x_{1},x_{2},...,x_{d})=g_{1}(x_{2},x_{3},...,x_{d-1})$, and Type 2, defined by $f(x_{1},x_{2},...,x_{d})=g_{1}(x_{2},x_{3},...,x_{d-1})+(x_{1}+x_{d})g_{2}(x_{2},x_{3},...,x_{d-1})$. For each type, we presented counterexamples (when *d* = 3) demonstrating that, in general, these local rules do not always produce *F*-squares. This highlighted the necessity to further investigate which forms of Type 1 and Type 2 local rules do guarantee equal row and column sums. Our findings revealed that, for Type 1, when $g_{1}(x_{2},x_{3},...,x_{d-1})=A_{0}$, where $A_{0}\in {𝔽}_{2}$, and for Type 2, when $g_{2}(x_{2},x_{3},...,x_{d-1})=1$, the required condition achieved. These specific families of local rules correspond to the constant local rules and bipermutive local rules, respectively, leading to the conclusion that the *F*-squares generated from CAs over ${𝔽}_{2}$ are either trivial *F*-squares or Latin squares.

From a practical point of view, this result is still informative for applications in experimental design. The works of Aggarwal et al. [10–13] and Husain et al. [14–17] show that *F*-squares are used to construct a variety of mixture designs. Our analysis indicates that, when we restrict attention to CAs over ${𝔽}_{2}$, such designs can only be obtained from trivial *F*-squares or Latin squares. Since Latin squares and trivial *F*-squares are themselves valid frequency squares, this means that binary CA constructions can be employed as an alternative way to generate designs of the types considered in these studies, particularly when the number of treatments matches the order of the square. On the other hand, our results indicate that binary CA constructions naturally align with Latin-square-based structures already used in cryptography, such as those appearing in threshold secret sharing schemes and in the use of CAs to construct bent and correlation-immune Boolean functions and pseudorandom sequences [26].

An immediate avenue for future research is to explore the necessary conditions under which *F*-squares can be generated by CAs defined over arbitrary finite fields ${𝔽}_{q}$, where *q* > 2. For such CAs, the entries of the CA-generated square are symbols drawn from an alphabet of size $q^{d-1}$, obtained by encoding vectors over ${𝔽}_{q}$. In this case, each coordinate of a symbol can take *q* values which is greater than 2 (the binary case), and there are many more ways to distribute these symbols while still keeping their total frequencies equal across all rows and columns. This suggests that non‑trivial, non‑Latin *F*-squares could in principle arise from suitable CAs over ${𝔽}_{q}$. Based on the case of *q* = 2, we can conjecture that there exist at least *F*-squares of types $\left(q^{d-1};q^{d-1}\right)$ and $\left(q^{d-1};1\right)$. A natural next step is to examine whether the observed pattern persists for *q* = 3.

For ${𝔽}_{3}$, we consider the simplest diameter, namely *d* = 2. Let ${ℱ}^{\left(c\right)}:{𝔽}_{3}^{2}\to {𝔽}_{3}$ be a CA defined by the local rule $f^{\left(c\right)}:{𝔽}_{3}^{2}\to {𝔽}_{3}$ given by


$$\begin{array}{c} f^{\left(c\right)}(x_{1},x_{2})=\left\{ \begin{array}{cc} 1, & \text{if }x_{1}\text{≠}x_{2},\\ 2, & \text{if }x_{1}=x_{2}. \end{array}\right. \end{array}$$

**Fig 5:**
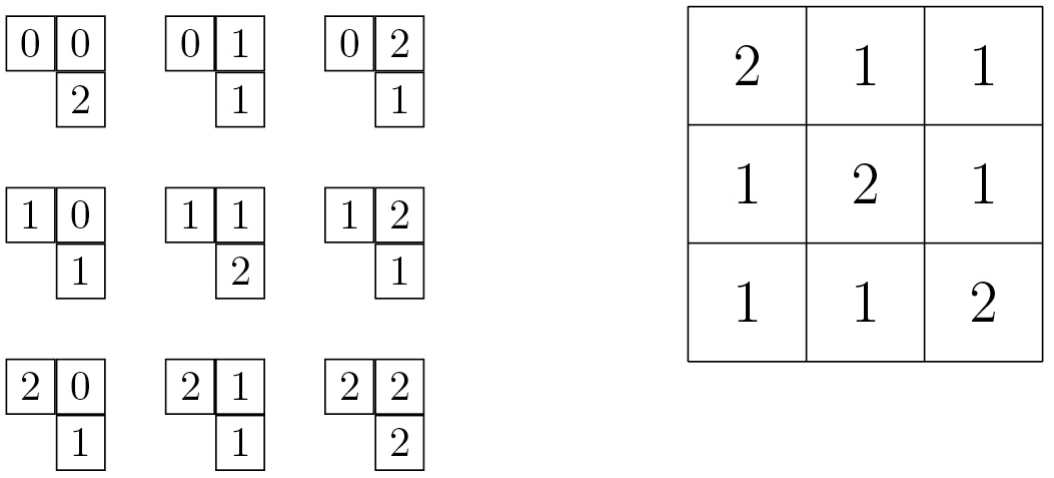
Truth table of ${ℱ}^{\left(c\right)}$ and the associated square ${S_{F}}^{\left(c\right)}$.

The associated square, shown in Fig 5, is an *F*-square of type (3;2,1), and it is neither a Latin square nor a trivial *F*-square.

This example illustrates that, for *q* = 3, the local rule acts on a larger alphabet, and the equal-frequency condition on rows and columns can be satisfied by symbol distributions that are neither fully Latin nor trivial. It is therefore plausible that further non-trivial, non-Latin *F*-squares might exist for CAs over ${𝔽}_{3}$. This investigation could benefit from a formal definition of potential non-trivial, non-Latin, *F*-squares. The ultimate goal would be to determine whether such *F*-squares exist and, if so, to characterise their structure and corresponding local rules. This generalisation introduces new structural and algebraic challenges, particularly in understanding how the local rule behaves over a larger alphabet and how it influences the combinatorial properties of the resulting squares.
